# Wild data: how front‐line hospital staff make sense of patients’ experiences

**DOI:** 10.1111/1467-9566.13115

**Published:** 2020-05-31

**Authors:** Catherine M. Montgomery, Alison Chisholm, Stephen Parkin, Louise Locock

**Affiliations:** ^1^ Centre for Biomedicine, Self and Society University of Edinburgh Edinburgh UK; ^2^ Nuffield Department of Primary Care Health Sciences University of Oxford Oxford UK; ^3^ National Addiction Centre Institute of Psychiatry, Psychology and Neuroscience King’s College London London UK; ^4^ Health Services Research Unit University of Aberdeen Aberdeen UK

**Keywords:** patient experience, quality improvement, data practices, ethnography, England

## Abstract

Patient‐centred care has become the touchstone of healthcare policy in developed healthcare systems. The ensuing commodification of patients’ experiences has resulted in a mass of data but little sense of whether and how such data are used. We sought to understand how front‐line staff use patient experience data for quality improvement in the National Health Service (NHS). We conducted a 12‐month ethnographic case study evaluation of improvement projects in six NHS hospitals in England in 2016–2017. Drawing on the sociology of everyday life, we show how front‐line staff worked with a notion of data as interpersonal and embodied. In addition to consulting organisationally sanctioned forms of data, staff used their own embodied interactions with patients, carers, other staff and the ward environment to shape improvements. The data staff found useful involved face‐to‐face interaction and dialogue; were visual, emotive, and allowed for immediate action. We draw on de Certeau to re‐conceptualise this as ‘wild data’. We conclude that patient experience data are relational, and have material, social and affective dimensions, which have been elided in the literature to date. Practice‐based theories of the everyday help to envision ‘patient experience’ not as a disembodied tool of managerialism but as an embedded part of healthcare staff professionalism.

## 1 Introduction

Over the past decade there has been increased focus on ‘the patient experience’. In the UK, this has been catalysed by high profile scandals in the National Health Service (NHS) and their attendant inquiries and reports (Francis 2013, Kirkup 2015, National Advisory Group on the Safety of Patients in England 2013). ‘Patient‐centred care’ has become the touchstone of healthcare policy – not only in the UK, but in other developed healthcare systems also, leading to what Sheard *et al. *(2019) characterise as ‘a zeitgeist moment’. Reflecting this, a patient experience ‘industry’ has grown up, spawning new technology, conferences, journals, dedicated job roles and departments, and consultancy firms whose business is to harvest and package data for healthcare‐providing organisations. The ensuing commodification of patients’ experiences (Lupton 2014, Mazanderani *et al.* 2013) within new logics of accounting and accountability (Numerato *et al.* 2012) has resulted in plentiful data but little sense of whether and how such data are used. Indeed, some argue that in spite of this activity, over the past decade ‘there has been little change in measures that reflect a person‐centred approach’ (Flott *et al.* 2017).

Patient experience – alongside patient safety and clinical effectiveness – is a key component of quality of care. It is important both as an end in itself, and because positive patient experience has been shown to be correlated with other clinical and organisational outcomes. Improving patient experience is thus a priority for the NHS, which has led the way in developing measures of patient experience such as the NHS Inpatient Survey (Duschinsky and Paddison 2018). Since April 2015, all NHS patients who have attended a healthcare facility in England have been invited to report back on their experiences, whether through the so‐called ‘Friends and Family test (FFT)’ (‘how likely are you to recommend our service to friends and family?), surveys, or narrative methods. The data that are collected are what are known as ‘patient experience data’. In spite of the quantities of data that are now collected, there is little evidence that these data are leading to improvements. There is a need to move beyond *collecting* patient experience data to *using* them to improve care (Coulter *et al.* 2014), but the evidence for the most effective ways to do this is weak. Specifically, we know little about how front‐line staff make sense of or contest the data, what supports or hinders them in making person‐centred improvements and what motivates staff to get involved in improvement work. The research reported in this paper was designed to fill this gap.

### 1.1 Sociology and ‘the patient experience’

A broad, sociologically informed literature exists which seeks to understand patient experience in the context of healthcare quality improvement (Flott *et al.* 2017, Martin *et al.* 2013, Renedo *et al.* 2018, Sheard *et al.* 2017). In a review of the challenges of using patient‐reported feedback to drive change, Flott *et al. *(2017) identified many factors, from staff scepticism about data quality to aggregation of data at organisational level which does not inspire local clinical ownership. Across studies, patient experience data have typically been assumed to be formal, organisationally sanctioned types of data (Dudhwala *et al.* 2017). Indeed, in spite of recognising that spontaneous interpersonal exchanges between individual patients and healthcare professionals are used by ward staff to improve care, researchers have limited themselves to analysing formalised sources of patient feedback (Sheard *et al.* 2019).

Outwith the focus on quality improvement, a more critical body of work directs us to think about what ‘patient experience’ actually refers to, how it has come to operate as a matter of concern and how it comes to be captured, codified and circulated through knowledge‐making practices.

For example, rather than accepting at face value that staff are sceptical about the *quality* of data, research suggests that we should be wary of assuming that patient experience is a stable or given epistemic resource (Pols 2005, Ziewitz 2017). Pols’ work points us in the direction of analysing practical matters rather than perspectives, attending to how meaning is co‐produced between people in specific material encounters. In attending to patients who cannot speak, Pols demonstrates how nursing staff nonetheless come to know their patients’ preferences through enacted, albeit tacit, situations. Subjectivity, she suggests, is related to situations rather than to individuals – directing our attention beyond the ‘authentic’ experience of the individual (as ‘captured’ by surveys and other measuring tools) and towards material environments and social interactions.

Likewise drawing attention to the embodied nature of data, Mazanderani *et al.*’s (2012) analysis of how illness experiences come to be valued as sources of health‐related knowledge found that the medium through which experiences were articulated and shared (such as text, image, voice or bodies) was central to how they were appropriated. Although their study focused on the meaning created between patients who shared a particular health condition, their findings are relevant for the way in which patient experience data come to be valued as a source of information for quality improvement by front‐line staff. Drawing on Abel and Browner (1998), Mazanderani *et al*. found that ‘others’ experiences would not be considered knowledge if they were not deemed, in some way, as an empathetic (shared) embodiment’ (p. 551). The focus on embodiment is important here; the authors observe, ‘people’s bodies serve as important vehicles for the articulation of experience, which means that the visibility of patients’ bodies plays a significant role in the sharing of experiences’ (p. 551). In relation to our own findings, how experience is mediated and its affective impact are both important dimensions to consider.

While these authors demonstrate the importance of embodied and affective data, other studies suggest that this kind of information – so‐called ‘soft data’ – is not easily used by healthcare organisations (Martin *et al.* 2015, 2018). Martin *et al. *(2015) describe soft data as ‘the kind that evade easy capture, straightforward classification and simple quantification’ (p. 19) and report that participants in their study – senior leaders of health systems in England – identified soft data as providing ‘rich, detailed, specific and highly pertinent insights into real or potential problems in quality of care’ (p. 22). Nonetheless, making sense of such data – turning them from data into intelligence – proved challenging and ultimately problematic, since the processes involved ultimately stripped soft data of all that was valuable about them in the first place (Martin *et al.* 2015). Martin *et al. *(2018) reinforce these findings in a subsequent study, in which they show that the very managerial mechanisms designed to render soft data useful may inadvertently silence them at source.

As outlined above, the literature on making sense of patients’ experiences of health care has included two important theoretical moves. Firstly, it has provided us with a practice‐based approach which examines the co‐production of subjectivity and the enactment of ‘appreciations’. This takes us beyond an analysis of the patient perspective but does not focus on the structural relations within which practices occur or, more specifically, how patient and staff experience of healthcare have been defined by politicians, policymakers and managers and how this relation itself informs practices. Secondly, the literature has suggested that data – which may be ‘soft’ or ‘hard’ – need to be ‘identified, selected, processed, interpreted, and made the basis of action’ in order to be rendered useable in healthcare organisations (Martin *et al.* 2015: 20). The focus of this body of work has been on how *managers* can derive meaning from soft data. What is missing is the link between these two approaches, which would render visible, and explain, how front‐line staff are in a constant process of co‐producing experience data with patients and creatively responding to such data outwith the need for translation or managerial sanction – something we observed empirically in the study reported here.

A re‐evaluation of the sociology of everyday life, drawing on the work of Michel de Certeau, helps us analyse this activity as both part of the everyday experience of working on a hospital ward, and as an act of creative resistance in the face of institutional pressure to engage in formal and narrowly metricised quality improvement work. Returning to the original language of de Certeau also provides us with a vocabulary with which to move beyond the terms ‘soft’ and ‘hard’ to consider these data as ‘wild’, that is lively, untamed and powerful.

### 1.2 Patient experience as a feature of ‘the everyday’

The sociology of the everyday has been a neglected resource in making sense of feedback in healthcare. While for patients, providing formal feedback may be an exceptional event, for front‐line NHS staff, interacting with patients and responding to them is a ubiquitous and mundane part of their everyday practice.^1^ To date, this activity has not been seen as part of patient experience work, a distinction which our analysis, informed by the work of Michel de Certeau, challenges.

de Certeau has been influential in the sociology of everyday life, which contributes to understandings of how ‘the familiar is significant as a dynamic site of social practice and exchange’ (Neal and Murji 2015). His work is relevant here because of its focus on everyday acts as sites of creative resistance – a theme which emerges in our own work when front‐line staff are tasked with doing quality improvement under routine conditions of extreme workload pressure. What characterises the everyday for de Certeau is a creativity and inventiveness which people enact from within a dominant economic order imposed from above (Highmore 2001a). In *The Practice of Everyday Life*, de Certeau looks at how people re‐appropriate dominant cultural forms in everyday situations in order to make them their own (de Certeau 1984). By focusing on the realm of routine practices, de Certeau shows how ordinary people engage in acts of creative resistance to the structures imposed upon them. To do so, he distinguishes between strategies and tactics. Strategies are the preserve of those operating within organisational power structures and are used to institute a set of relations for official ends. Tactics, by contrast, are used by those who are subjugated; they occur in spaces produced and governed by more powerful strategic relations, and are therefore opportunistic and momentary: ‘a tactic . . . is always on the watch for opportunities that must be seized “on the wing”’ (xix). Particularly relevant for our own investigation is de Certeau’s focus on the relation between producers and users of material culture. In terms of quality improvement, the relation between the producers of patient experience *data* and its users has rarely been examined, nor has the extent to which such data are material.

In our own investigation, the focus was on front‐line staff as the users of patient experience data, but also on the *ways* they used data and the *characteristics* of data that made them credible and useful. While drawing on the notion of strategies and tactics to examine the former, we also take up de Certeau’s trope of the wildness of the everyday to explore what it was about data that made them useable for staff. Highmore writes:

For de Certeau the ‘wildness’ of the everyday resonates in both a major and minor key. At one level, the wildness of the everyday is simply the ‘untamed’: it is what gets remaindered when the everyday is scrutinised from a rationalistic perspective (major key). It is also, more mundanely (and more appropriately), all those burps, hisses, whispers, crackles and slurps that sound engineers refer to as ‘wild’ and that get filtered out in the production process of sound recording (minor key) [. . .] ‘Wild things’ [. . .] are the unwanted, unanticipated, extraneous, excessive meanings that have to be filtered out in accounts of objects. (Highmore 2001b)

We find a parallel to this in the production of patient experience data collected through organisationally sanctioned surveys. Through this process, a patient’s experience is filtered down into neat, predefined categories, while the ‘burps, hisses, whispers, crackles and slurps’ – which staff may absorb through their daily interactions with patients and carers – are filtered out. These ‘wild data’, we show, can be a source of staff creativity when it comes to improving patient care. Combining these two strands of de Certeau’s work, we examine how front‐line staff made sense of patient experience data in the context of their everyday work.

## 2 Methods

As part of a National Institute for Health Research‐funded study we undertook an ethnographic case study evaluation of how front‐line staff use patient experience data for quality improvement in six NHS hospitals in England. The year‐long ethnography was part of a larger study (Locock *et al.* 2020a, 2020b). Case study sites were purposively selected to reflect a range of contexts, from organisations which were performing well on staff and patient experience measures and had a strong track‐record on quality improvement to those facing organisational challenges and where person‐centred improvement was less embedded. They included various types of ward: two general medical wards, a gastric medicine ward, two emergency medical assessment units and a longer‐term rehabilitation medical ward. Geographic diversity was achieved by including both urban and more mixed ‘town and country’ catchments, and covering north, south, east, west and midlands locations across England. All sites are anonymised, in line with our ethics approval and to ensure participants felt comfortable sharing more negative views and experiences.

Each site nominated a medical ward to take part; a team of front‐line staff from each ward attended a 2‐day learning community organised by the research team, and led their local improvement work. Three ethnographers observed the teams over 1 year and conducted interviews at several time points. The data drawn on in this paper consist of 95 in‐depth interviews with front‐line staff and senior managers, as well as almost 300 hours of observation. We observed learning community events, local quality improvement planning meetings, meetings of patient and carer experience groups, general staff meetings and workspaces, supplemented by informal conversations with staff. Observations were guided by a shared pro‐forma. Data collected included written fieldnotes, individual reflective notes, documents and photographs (e.g. of comments boards or information displays prepared by front‐line teams as part of their work). The nature and amount of observation varied by site, depending on front‐line staff’s chosen improvement activities, and was affected by severe workload pressures in the NHS during winter 2016–2017.

The three ethnographers analysed the ethnographic data in NVivo 10/11 (QSR International Pty Ltd. Version 11, 2015; Version 10, 2014) using a shared, inductively generated coding framework, which included: types of patient experience data used; attitudes towards/understanding of data; team composition and membership; relationships with Patient Experience Office and senior management; organisational pressures and constraints. Thick case descriptions were produced for each site, along with process maps, as part of a comparative thematic analysis. Ethics approval was obtained from the NHS Health Research Authority (North East – York Research Ethics Committee: Ref. 16/NE0071).

## 3 Findings

### 3.1 Into the wild

We started this study with the expectation that staff would draw on formal types of patient experience data (both quantitative and qualitative, from surveys to observation) to guide their quality improvement interventions. There were varying degrees of experience and expertise in patient experience across the different sites, with some purposively selected due to their strengths in this area. Nonetheless, our twofold assumption – that the meaning of patient experience data is clear and that they are used by staff – proved flawed almost immediately when it became apparent that not all staff had heard of patient experience as ‘a thing’; not all staff knew what patient experience data were or what kinds were collected in their hospital; and staff did not necessarily use patient experience data, as normally conceived by the NHS, in their improvement projects.

The reasons why staff did not necessarily – or even primarily – use formally recognised patient experience data in their improvement projects were not articulated straightforwardly, but surfaced in accounts of the structures and hierarchies within which staff worked. It was clear that in some sites, formally encoded patient experience was being used to performance manage staff punitively. In the extract below, patient experience is tied up with audit and admonishment. It is far removed from patients.

Matron: Every month we get hauled up in front of a board of hierarchy with our performance reviewed. And the patient experience group deliver audit detail [. . .] So someone might say, “Oh, [ward name], you’ve only got 30‐, you know, 32 per cent of people have replied” and this, that and the other and, “It’s all around discharge.” So that seemed to be every month what I was getting told off for. Well, not told off but, you know, reminded about. And so we tried to put a few things in place, things like changing the time of day that we gave out the questionnaire to the patients and things like that. (1st interview)

Rather than seeking to improve patients’ experiences, the matron focuses instead on improving the response rate (by handing out questionnaires at a different time of day) – seen as a proxy for improving the data. Asked about how things had improved, she went on to say ‘I’ve gone into green now for 2 months running’.

Referencing the RAG rating system – using the traffic light colours red, amber and green to rate issues – the matron alludes to the hinterland of patient experience against which staff targets are set. Within this context, ‘patient experience data’ refers to nationally mandated surveys, such as the FFT, and the more statistically reliable NHS inpatient survey. Measures such as the FFT have been subsumed into the government framework of CQUINs (Commissioning for Quality and Innovation), whereby a proportion of healthcare providers’ income is conditional on demonstrating improvements in quality. Rather than rewarding hospitals for substantive improvements, these have sometimes been focused on response rates, generating a culture of measurement rather than action (Bailey *et al.* 2019). In a first interview about her role, the Head of Patient Experience at one case study site alluded to the way in which CQUINs had shaped patient experience data collection:

When we realised that out‐patients was on the horizon and it was obviously a CQUIN target, so we got paid money for it, a lot of money for rolling it out, I obviously had to go out to tender for a company to do that for us [. . .] and actually the CQUIN was for three hundred and ten thousand pounds, and it cost us fifty thousand pounds to get a company in, so it was worth it. I have never, ever missed a CQUIN ever for response rates, anything; I've always met my CQUINs so it's . . . yeah and again that was another thing that kept pushing it higher up the agenda, because it was actually generating money. (1st interview)

The outsourced, impersonal, income‐generating version of patient experience described above corresponds to the dominant organising frame within which patient experience is operationalised in the NHS. In de Certeau’s terms, it represents the strategic interests of those who control the means of cultural and economic production. By contrast, front‐line staff in this study worked with a notion of data as material, interpersonal and/or embodied. Rather than seeking out the organisationally sanctioned forms of data, several teams went about generating new data through person‐centred activities and physical artefacts which could be incorporated into their daily practice. One site placed a ‘bubble board’ on the wall with blank paper speech bubbles for patients to write their thoughts and feelings on. Ward staff encouraged patients and visitors to fill in a bubble, an interaction which became as much an intervention in demonstrating care as a way of collecting data:

Healthcare assistant: We had one lady that, she'd had some really sad news, and just sitting with her for 5 minutes, chatting to her; she didn’t want to chat about what she was upset about. So, just chatting to her and saying, "There's a bubble there for you if you want to write anything down," and she just wrote a little something. I think it was just a face with a little sad mouth, and that was enough for her. So, I think it just takes something off of someone's shoulders [. . .] Since we've been doing this, people are recognising things I think a little bit more, and I think that’s what's changing; not the bubbles on the board and things like that. It's recognition that somebody needs to have a good experience. (2nd interview)

Similarly, another site devised a ‘What Matters to Me Tree’, where staff, patients and visitors could write feedback. The ward manager explained how it seemed more tangible and meaningful to staff than traditional audit data. The physical data collection artefact became itself a motivator, an emotional and enjoyable representation of care.

While the sites differed in the extent to which they drew on traditional patient experience data, there were many examples of staff using their everyday interactions with patients, carers, other staff and the ward environment to shape improvements. These interpersonal exchanges led to changes in the ward environment and patients’ experiences of a stay in that environment. They transformed the ward from a managerially organised place to a space shaped by the lived experience of its occupants. What is significant in these examples is not that they were ground‐breaking strategic investments in improving *the* patient experience, managerially conceived, but to the contrary, that they were ‘tactical’ responses (in de Certeau’s terms), suggested by patients and implemented by front‐line staff, which made a difference in the here‐and‐now. They included:

1. A welcome pack for patients based on staff’s observation that many patients came in without any personal effects
2. Hearing aid boxes for patients based on staff’s experience of the distress lost hearing aids caused to patients
3. Installation of new cupboards, which staff knew would improve their responsiveness to patients based on the layout of the ward
4. A fix for a squeaky bin
5. A request that the kitchen serve jelly on cold plates rather hot plates, so that it wasn’t a melted pool by the time it reached patients

When asked how the team had the idea for a welcome pack, one consultant said: ‘It fits right, but I don’t think they’ve done a survey’. At another site, the ethnographer probed where the inspiration for the hearing aid boxes had come from:

Interviewer: Things like the hearing aid boxes I think came from the staff suggestion board, didn’t it, rather than from the patient experience data?

Ward manager: Yeah, all of that just came up because we could see the distress . . . of the patients that have lost an expensive hearing aid and they can't communicate at a time when they most need to communicate clearly. . . . That came from, you know, our own experience of what causes them distress. (2nd interview)

Initially, we were disappointed that the ward teams were not following the project brief asking them to use patient experience data. Not only that, but some of the reinventions of everyday work spaces and relations seemed rather unambitious – too mundane – and were not as top‐level or strategic as we had hoped for. Although we had started with a broad and inclusive definition of patient experience data, which included everything from surveys to comments on social media, public meetings and patient stories, as the ethnography progressed, we began to realise that our very conception of ‘data’ was at issue.

### 3.2 Wild data are ‘real data’

Describing the kind of data they liked or found useful, staff at different sites used the word ‘real’ and said they involved face‐to‐face or embodied interaction, allowed for dialogue between staff and patient, were emotive, and allowed for action. Ultimately, ‘real’ data were immediate in all senses of the word, both temporally and bureaucratically unmediated.

Junior ward sister: I like real data because I think real data is spontaneous and you can try and act on it as soon as you can and that can make a change for the next person coming through that door. It's quite active data. (1st interview)

At a second site, a staff member voiced the same sentiment, highlighting the value of co‐presence in constructing patient experience data:

Ward manager: But when you actually listen sometimes to the patients saying you know, “I felt, this made me feel really,” and you think, “Oh gosh,” You know this is real, they’re not being confrontational, they’re actually expressing being really unhappy about something, and *it’s different hearing it than it is seeing it written*. (1st interview, emphasis added)

Actively ‘being there’ with the patient, rather than passively receiving an abstracted measure, was important to staff, as was being the feedback instrument itself. These small acts of everyday practice, which de Certeau characterises as resistances, challenged the institutional rationality of patient experience instruments and quality improvement strategies in ways which sometimes provoked discomfort amongst managers and other colleagues.

For example, at another site, an activities coordinator, who was very active in the improvement project, had – in his own words – ‘created this big fancy timetable for the ward in terms of activities’. A more senior member of the team had suggested taking the timetable down, as he recounts here:

Activities Co‐ordinator: [Head of Quality Improvement] said, “I know [name]’s enthusiastic but actually we need to take this down because it hasn’t come from patients and it hasn’t come from, you know, carers and family members and hasn’t even had an input with staff.” But it had, because I’d already went around them and, “What do you think?” or, you know, “What can I do for you the day?” to the patient, to the staff, “What do you think? Do you reckon that would work?” Because you, you’ve been looking after them for the last ten days. “What do you think?” “Yeah, yeah, it would work.” So it was co‐design, just in, not around the table in a structured, mapping out kind of way. (1st interview)

This account raises several dimensions which we saw across our data, such as the importance of recognising the link between staff experience and patient experience and the value of informal, unsanctioned, and unformalised kinds of feedback. As well as actively engaging with patients to make improvements on the ward, the activities coordinator was making video blogs (vlogs) about his experiences at work. When asked how the vlog would inform improvements on the ward, he replied:

It’s real, it’s honest, it’s transparent, it’s not hiding away from anything. And it’s real time. It’s not something we’re gonna sit down in a year’s time and say, “Actually on week two [um] I felt this way.” Cos we’re probably gonna, might just forget that. Or overshadow actually the real moment and the real emotion. But I find if we’re recording it now in real time and that emotion is raw, I think we’ll get a real perspective. (1st interview)

‘Real’ data traces its roots to the Latin *res* meaning matter or thing. Real data are concerned with the materiality of care; they make the connection between the material and the authentic. They are opposed to what is abstract, decontextualised, immaterial – and sometimes, therefore, irrelevant. In de Certeau’s terms, real data, as described by staff above, represent creative resistance to the structures imposed upon them by their organisations’ Patient Experience and Quality Improvement strategies. These trade in spreadsheets, dashboards, RAG ratings and implementation plans. Making use of embodied interactions on the ward is a tactic, an opportunity ‘seized on the wing’ within the constraints of high work demands and a pressured environment, where the time and space to engage with managerial instruments is often non‐existent. Reflecting this disconnect between patient experience as a relational achievement between front‐line staff and patients, and patient experience *data* as something produced and curated in hospital back offices, are the following extracts:

Ethnographic fieldnote: The issue of the link between patient and staff experience was raised and discussed by the group, and how we know there’s a link but don’t know the direction of causality. [Name] came back to language of: capture, measure, balanced scorecard. (Observation of Patient Experience Strategic Group)

Consultant: I think even for us who work in the NHS, the middle management are like a grey fog. I’m not sure exactly what most of the people do here and, you know, how many layers you have before you get to the person who can make the decision. (1st interview)

In understanding patient experience data as produced through their own daily practices on the ward, front‐line staff themselves experienced an increased willingness and capacity to act on improving care.

### 3.3 Capturing wild data

So far, we have described wild data as the informal, embodied and sometimes intuitive knowledge about patients’ experiences that staff acquired through daily interactions on and with the ward. Patients’ stories, compliments and other material expressions of experience, such as thank you cards and boxes of chocolates, were also discussed by staff, but fall outside what is normally referred to as patient experience data. However, hospital managers were starting to recognise these less obvious forms of data – sometimes referred to as ‘soft intelligence’ – as evidence that needed to be captured and quantified, as exemplified below.

Consultant: I think the ward managers are asked to keep a log of any compliments, and then . . . it sounds really crass – boxes of chocolates – count the number of boxes of chocolates you have and thank you cards. (1st interview)

The Head of Patient Experience in the same organisation expanded on this:

Head of Patient Experience: So, compliments . . . if the Chief Exec, for example receives a letter, a compliment letter, then that will be captured; we've got an internal reporting system called Safeguard. So, we would scan that letter in, log it on Safeguard; that compliment has been captured [. . .] on a monthly basis the number of compliments we've captured gets reported to leadership brief [. . .] What the divisions and the departments and wards do, at ward level currently, is complete a crib sheet. So, when a compliment is received, whether that’s via a gift, whether it's via a written card, telephone conversation, face‐to‐face conversation, the staff will just tick box a crib sheet and we'll record that as well. (2nd interview)

In another site, there was a similar emerging focus on ensuring that all forms of feedback were accounted for through formal reporting mechanisms. The fieldnote below describes one instance, during a meeting of the sub‐Board committee relating to patient and carer experience:

One of the main points she [Head of Patient Experience] emphasizes is that there is currently no process for storing patient stories, and this is something they need to develop. She references the RCN Leadership Programme, which ward managers will be undertaking. She says they will be teaching people to capture patient stories and will be expecting ward managers to capture patient stories. We “should be giving sugar lumps” when they’ve done well, but also capturing lessons learned. There’s a lot of use of the word ‘capturing’ and a certain breathlessness imputed to this activity, as if patient stories might otherwise escape. (Observation of Patient and Carer Experience Group)

It was not only positive feedback which was subject to such managerial processes of capture, storage and report. In an observation of one site’s Patient Experience Strategic Group, the ethnographer noted under the agenda item ‘Complaints report’: ‘Discussion of “feedback dashboard” and “Balanced scorecard” and “PMF” (performance monitoring?)’. In another, the ethnographer noted:

It’s interesting to me that ‘learning from complaints’ is a thing, and a thing that can be captured and put in a database. Clearly, it is not enough that people learn from experience, but – as [Director of Nursing] says – that there is evidence that learning has occurred. Speaking about learning as a *thing* rather than a *process* has the effect of petrifying what should be a living and dynamic action. (Observation of Patient and Carer Experience Group)

While accountability is an important constituent of care, when it is reduced to an exercise in accounting, as described above, its effects are diminished. Recording that learning has occurred is not problematic per se, but in this case was indicative of a culture of capture superseding a culture of action.

In the national context of hospital failings, at some sites the use of patient experience to performance manage staff led both to a ‘fossilization’ of wild data, and to a sense of threat:

Ward manager: When you see a complaint on paper you don’t relate it to that person, and it feels very threatening. (1st interview)

The need to document everything was in some cases deflecting attention away from giving good care to ‘capturing’ that it had happened, as the following fieldnote illustrates:

End of Life Project Lead speaks about getting feedback from bereaved carers. “We should be capturing this information.” Findings are fed up to the strategy group and down to the operational group. Dying person’s care plan – everyone who’s dying should have one, but figures fluctuate and aren’t very good. “That’s not to say they’re not receiving the care, but it’s about the documentation of that care.” (Observation of Patient and Carer Experience Group)

In this meeting of the patient and carer experience group, the logics of accountability and the logics of care are coupled. While both systems of accountability (‘strategy’) and the spontaneous and relational dimensions of care (‘tactics’) are important to ensuring patient wellbeing, in some sites, the former was at risk of eclipsing the latter and devaluing them as the currency of patient experience. This was not the case everywhere; in one site, organisational approaches to managing patient experience gave staff free rein to be creative and respond to wild data on the ward.

### 3.4 Channelling data

Data practices are part of a politics of power within the hospital. Things on the ward that produce data are in many cases about professional control over nurses (e.g. VitalPAC, a mobile software information system for monitoring patients’ vital signs). When given the means to generate their own data, front‐line staff produce something very different, which expands rather than constrains their creativity. Below we see how front‐line staff envisioned physical and empathetic proximity to patients as the best means of understanding their experiences. Rather than ‘capturing’ information, this was about experiencing *with* patients through embodied interactions and activities, such as experience‐based co‐design, talking to patients at the bedside or consciously putting themselves in a patient’s shoes.

Ward manager: Doing the experience‐based co‐design, it was about sharing and owning something together and working together to achieve something. (2nd interview)

Staff nurse: When you do your training it's all very regimental – this is what you do, this is how you do it. And then when you come to work it's the same. So, you come in, you do your washes, you do your meds, do your pills, you do beds, you do this, you do that, and then you do your notes and then you do it all over again. It almost seems like there's no time for anything else [. . .] But actually there is, and it's just finding the time [. . .] “We've got half an hour here, let me sit down, let me have a chat with the patients, see how they're getting on [. . .] It is just sitting down and having that extra 5 minutes that they appreciate [. . .] It does make you more conscious. It's less task‐focused and more patient‐focused, which is what the project is about. (2nd interview)

Ward manager: You need to put yourself in the place of the person who’s having the treatment, and any way that that can be done, either by sitting and listening to somebody, or being a patient, or just having time to think how you might feel if you were being bed bathed with a curtain, where somebody’s pulling the curtain open and saying, “Gladys, do you want another cup of tea?” when you’re there half naked, you know. It’s so part of the environment to us, you’ve really got to re‐think and step back, and anything you can do to make people feel they are in that place, and to be looking from the inside out instead of looking from a nurses’ uniform at this, a patient. I love it because I think that’s the most powerful thing. (1st interview)

For some of the staff taking part in this project, engaging with the concept of patient experience reconnected them with the idea of person‐centred rather than task‐focused work. It also became apparent to staff that working with patient experience data could incorporate their daily interactions with patients and everyday objects of care on the ward – i.e. that improvements in patient care were part and parcel of the material culture of their workplace. Oftentimes, staff felt they did not need a survey to tell them this; much of what they felt needed improving was already apparent to them. Dialogue between front‐line staff and those working in the patient experience office in some cases led to a recognition of this at a managerial level, as the extract below illustrates:

Head of Patient Experience: *Staff who are close to their patients can see the best*. You know, this survey is very much reliant on people who can fill it in. So, actually, you've got lots of other patients that you’re perhaps not considering when you just take that data. And it's also very lengthy. When you look at all the questions, you know, I am bored after ten questions, let alone, I think there's 87 or 89. Whereas some of the dementia patients might not be able to tell you, but *you can see just by doing certain activities or things with them*, that made a difference to them. (1st interview, emphasis added)

Front‐line staff can themselves be the instruments and repositories of data about patients’ experiences. As the study evolved, so too did an understanding of this amongst both the hospital staff participating in the research and amongst ourselves as researchers.

Patient Experience Officer: It depends how we look at data. I think in the ward staff before, if you said to them, “What's patient experience data?” they will say “Surveys.” I'm saying to them now data is any feedback at all, wherever that's coming from and in whatever form, whether that's coming from focus groups from the patients or anecdotal feedback from staff and patients, it's all patient experience data. (3rd interview)

### 3.5 Wild is dangerous

There were exceptions to those who felt empowered and enthusiastic about identifying patient concerns through staff rather than from formal patient experience data. While institutionally sanctioned data tended to present staff with impersonal survey results, ‘soft intelligence’ such as patient narratives and embodied interactions could feel too emotive and threatening. This was particularly so when negative feedback was perceived to be too direct or personal, unable to be captured or contained, or demotivating for staff. Some staff retreated to the safety of quantified, aggregated, abstracted and anonymous data fed down from above through managerial processes. Even within the same staff members, there could be a desire for ‘real’ data that was ward‐relevant and immediate, yet ‘safe’, simple and thematically organised for easy use.

Discomfort about expanding the definition of patient experience data to include staff knowledge and intuition about care was not limited to front‐line staff, but was discussed both amongst the research team and by some managers within the case study sites. There was apprehension that staff perspectives could be privileged over patients’ (particularly if the two diverge), that staff perceptions may not be a reliable guide, and that the progress made in listening to patients could be reversed.

Head of Patient Experience: See, for me I'd want to say, "Well where's the evidence? Where's the evidence to substantiate that?" and, "Yes, you might be right and I might agree with you," or, "Yes, I agree with you and we can back this up because of this, or this, or this, or this." But without any evidence, or something tangible to substantiate that, I think we can – not make mistakes – but we can go wrong in thinking we know what patients want. (2nd interview)

## 4 Discussion

In this paper, we have argued that ‘patient experience’ is a relational achievement, involving the interplay of people, places and things. It follows that patient experience *data* are relational, and have material, social and affective dimensions – which have largely been elided in the literature to date. Inspired by de Certeau, we have re‐conceptualised patient experience data to include ‘wild data’, to draw attention to these other qualities, which we argue can affect how front‐line staff engage with quality improvement based on patient experience. de Certeau’s insights into how ordinary people resist organisational power structures by re‐appropriating images, products and space to their own interests led us to consider front‐line staff not merely as the users of patient experience data, but also as their co‐producers. This complicates official patient experience strategies within NHS organisations, which, we observed, tend to value accounting systems (in the name of accountability) over spontaneous practices of care which may be less amenable to capture. de Certeau’s work helps us articulate how staff used everyday encounters on the ward to supplement and/or substitute officially sanctioned accounts of patient experience at organisational level.

### 4.1 Rewilding patient experience data

Rewilding refers to restoring ecosystems through the (re‐)introduction of species to their original habitat. In the patient experience industry, staff and staff experience have tended to be removed from what counts (or is counted), in spite of increasing evidence that patient and staff experience are linked (Dawson 2018, Maben *et al.* 2012, Sizmur and Raleigh 2018). This is hardly a surprising finding, since it is the *relational* aspects of care that matter most to patients, and caring as a process is inherently interpersonal (Ihlebæk 2018). Informed by the practice‐based orientation of sociologies of the everyday, our findings suggest the importance of re‐introducing staff’s embodied experience into the patient experience ecosystem. This can take various forms. One important way is to take seriously the tacit, intuitive, informal and embodied information – what we term ‘wild data’ – which front‐line staff encounter and produce in their everyday practice. This foregrounds aspects of experience which are frequently muted in the formal collection of survey data, such as the social and material dimensions of human experience. Attending to these aspects sheds light on something which often escapes capture in closed response questionnaires, namely culture, for as Graves‐Brown observes, ‘culture exists neither in our minds, not does it exist independently in the world around us, but rather is an emergent property of the relationship between persons and things’ (Graves‐Brown 2000).

A potential criticism of this approach is that culture and intuition are tacit and elusive and cannot therefore form the basis of experience ‘data’. Demystifying the way in which healthcare staff ‘intuit’ what patients are experiencing, Ihlebaek has analysed how nurses acquire and use their senses in everyday clinical practice, noting that ‘nurses’ expertise is cultivated in continuous, embodied, sensory, and intersubjective relations in the doing of nursing’ (Ihlebæk 2018). In her study of knowledge and professionalism among registered nurses at a cancer unit in a Norwegian hospital, she found that nurses ‘relied not only on what they themselves had sensed, but also on the patients’ accounts of their own bodily experiences, as well as relatives’ stories’ (*Ibid*: 493). The same can be true of healthcare assistants, ward clerks and other front‐line staff. Rather than characterising this professional knowledge as ‘soft’, or somehow inferior to the ‘hard’ data collected via formal patient experience instruments, we should see this expertise as a resource for improving care.

The sensory dimensions of patients’ experiences are rarely the focus of ‘the patient experience’ discourse, perhaps reflecting what Maslen – referring to doctors – identifies as ‘a gap between work ‘as imagined’ by policy makers and work ‘as done’ by doctors’ (Maslen 2016). Turning our attention to patients’ and staff’s experiences as *processes* which are at once social, material and affective could lead to a radical rethinking of how to better healthcare environments. This would entail a conceptual shift in data economies, from data‐based value creation linked to financial incentives to data as a cultural good exchanged in the pursuit of better care. Combining insights from sociologies of the everyday with some of the major concerns of medical sociology – such as power, inequality and the division of labour in clinical settings; hierarchies of knowledge and evidence in health care; and patient‐provider relationships – allows us to make the case for doing so.

While staff were able to act on wild data for the purposes of this research project, the extent to which the NHS as a whole can or should accommodate or even promote individual staff creativity remains a question to be answered. As noted above, everyday tactical responses by staff to structures designed to serve patient and hospital interests are likely to be seen as deviant and dangerous. One way to consider this is through the lens of organisational entrepreneurship, defined as ‘a form of social creativity . . . a tactical art of creating space for play and/or invention within an established order, to actualize new practices’ (Hjorth 2005). An entrepreneurial spirit has been advocated within the NHS (Godlee 2018) and research into institutional logics suggests the existence of partial autonomy for those working at the coalface (Checkland *et al.* 2017, Martin *et al.* 2016). Ushering in a shift from the era of assessment and accountability to an era of systems and creativity in the NHS, Black urges: ‘we need to accommodate and support social entrepreneurs, the creative disruptors who will instigate innovation’. He goes on:

Leaders must encourage and allow creativity to emerge by drawing together relevant people to tackle any given problem. This takes courage and insight because these people may not be in formal positions, such as medical directors, but be staff who in the past have had no voice. This is vital because creative solutions will reflect who is involved and the space they are afforded to think afresh. (Black 2018)

We have used de Certeau to describe front‐line staff precisely as the ‘creative disruptors’ Black calls for and shown how working with wild data alongside organisationally sanctioned data enables staff to engage in service improvement based on patients’ experiences.

It is important to consider that not all staff have the innate ability or capacity within the constraints of their working environment to observe, intuit, discern or act on what their patients are feeling and desiring. Nor would it be wise to assume that staff always ‘get it right’ when making judgements or assumptions about what patients want. We should be cautious not to privilege staff’s voices over and above those of patients, or inadvertently return to a culture in which ‘matron knows best’. However, we contend that expanding the patient experience lens to include practices not just perspectives, and sensory as well as survey data, can be a useful basis for understanding the constraints and possibilities around quality of care. Staff should be encouraged to be actively and imaginatively involved in improving the socio‐material life of the ward. Leaders should encourage a sensitivity for the practicalities of daily life and encourage staff both to be observant and to carefully check their own observations. In this way, we can move from ‘patient experience’ as a disembodied tool of managerialism to an embedded part of the professionalism which drives front‐line healthcare staff.

## Authors contributions

**Catherine M. Montgomery:** Conceptualization (supporting); data curation (equal); formal analysis (lead); investigation (equal); methodology (equal); writing‐original draft (lead); writing‐review & editing (lead). **Alison Chisholm:** Conceptualization (supporting); data curation (equal); formal analysis (equal); investigation (equal); methodology (equal); writing‐original draft (supporting); writing‐review & editing (supporting). **Stephen Parkin:** Conceptualization (supporting); data curation (equal); formal analysis (equal); investigation (equal); methodology (equal); writing‐original draft (supporting); writing‐review & editing (supporting). **Louise Locock:** Conceptualization (lead); formal analysis (supporting); funding acquisition (lead); investigation (lead); methodology (lead); supervision (lead); writing‐original draft (supporting); writing‐review & editing (supporting).

## Note

1 We are informed here by Robinson’s work reconceptualising the boundary between the mundane and the extraordinary: ‘the transformative capacity of the everyday itself has sometimes been overlooked by theorists, especially in relation to how the extraordinary is both embedded within and in dialogue with the mundane, rather than having a separate and unmediated existence of its own’ Robinson (2015).
